# Phenotypic similarity-based approach for variant prioritization for unsolved rare disease: a preliminary methodological report

**DOI:** 10.1038/s41431-023-01486-7

**Published:** 2023-11-06

**Authors:** David Lagorce, Emeline Lebreton, Leslie Matalonga, Oscar Hongnat, Maroua Chahdil, Davide Piscia, Ida Paramonov, Kornelia Ellwanger, Sebastian Köhler, Peter Robinson, Holm Graessner, Sergi Beltran, Caterina Lucano, Marc Hanauer, Ana Rath

**Affiliations:** 1 INSERM, US14 - Orphanet, Plateforme Maladies Rares, 75014 Paris, France; 2 CNAG-CRG, Centre for Genomic Regulation (CRG), The Barcelona Institute of Science and Technology, Baldiri Reixac 4, Barcelona, 08028 Spain; 3 Institute of Medical Genetics and Applied Genomics, University of Tübingen, Tübingen, Germany; 4 Centre for Rare Diseases, University of Tübingen, Tübingen, Germany; 5 Ada Health GmbH, Berlin, Germany; 6 The Jackson Laboratory for Genomic Medicine, Farmington, CT 06032 USA

**Keywords:** Genome informatics, Spinocerebellar ataxia

## Abstract

Rare diseases (RD) have a prevalence of not more than 1/2000 persons in the European population, and are characterised by the difficulty experienced in obtaining a correct and timely diagnosis. According to Orphanet, 72.5% of RD have a genetic origin although 35% of them do not yet have an identified causative gene. A significant proportion of patients suspected to have a genetic RD receive an inconclusive exome/genome sequencing. Working towards the International Rare Diseases Research Consortium (IRDiRC)’s goal for 2027 to ensure that all people living with a RD receive a diagnosis within one year of coming to medical attention, the Solve-RD project aims to identify the molecular causes underlying undiagnosed RD. As part of this strategy, we developed a phenotypic similarity-based variant prioritization methodology comparing submitted cases with other submitted cases and with known RD in Orphanet. Three complementary approaches based on phenotypic similarity calculations using the Human Phenotype Ontology (HPO), the Orphanet Rare Diseases Ontology (ORDO) and the HPO-ORDO Ontological Module (HOOM) were developed; genomic data reanalysis was performed by the RD-Connect Genome-Phenome Analysis Platform (GPAP). The methodology was tested in 4 exemplary cases discussed with experts from European Reference Networks. Variants of interest (pathogenic or likely pathogenic) were detected in 8.8% of the 725 cases clustered by similarity calculations. Diagnostic hypotheses were validated in 42.1% of them and needed further exploration in another 10.9%. Based on the promising results, we are devising an automated standardized phenotypic-based re-analysis pipeline to be applied to the entire unsolved cases cohort.

## Introduction

Rare diseases (RD) are defined in Europe as those affecting not more than 1/2000 persons in the European population [1]. 72.5% of RD have a genetic origin [2], although for 35% of them a causative gene has not yet been identified [3]. Rare diseases are characterised by the difficulty in obtaining a correct and timely diagnosis, because of their rarity, scarcity of patients and inequalities in access to expertise [4]. During their diagnostic journey, patients may receive a clinical diagnosis [5] (i.e., a name for their disease) or remain undiagnosed. For genetic disorders, identifying and characterising the underlying molecular basis is crucial for establishing a specific diagnosis and implementing an optimal therapeutic approach.

Solve-RD (Solving the unsolved rare diseases) [6, 7] is a European-funded research project that aims to molecularly solve unsolved cases defined as those without a molecular diagnosis after undergoing WES (whole exome sequencing), by using different data and re-analysis approaches [8] and with the ultimate goal of assigning a clinical diagnosis to yet undiagnosed patients [9]. Solve-RD builds upon a core group of four European Reference Networks [10] (ERNs: ERN-ITHACA, ERN-RND, ERN-Euro NMD, ERN-GENTURIS) which annually see more than 270,000 RD patients, and that contribute unsolved patients data. Amongst the approaches explored within this project, we present a methodology based on phenotypic similarity calculations among solved/unsolved cases (patients) and known RD. Indeed, it is well demonstrated [11–15] that using a phenotypic approach relying on phenotypic annotation comparisons is useful in gene prioritisation and diagnosis research [16–20], as for example Phen2Disease or LIRICAL, that compare patient phenotypes to annotated diseases. However, producing good quality phenotype annotations is burdensome for clinicians and there is room to test other approaches (i.e., Natural Language Processing, Artificial Intelligence, Machine Learning, Deep Neural Networks and entity recognition in clinical narratives) [15–20]. For the purpose of this study, we collected phenomic and genomic data from unsolved RD cases in a standardized and machine-readable format, and ran a series of one-to-all comparisons, including cases and known RD in order to raise diagnostic hypotheses based on re-analysis of candidate causative variants. These were then resubmitted to clinicians for further investigation and validation. Ultimately, this led to the identification of a formerly undescribed disease, or to the identification of unreported manifestations of known RD. The final goal was to return a clinical diagnosis to the patients. This article aims to describe the methodology used in our phenotype similarity-based pipeline by providing illustrative examples of its application.

## Materials and Methods

### Data management

The overall Solve-RD data management is documented [7, 8]. Briefly, after submission to the RD-Connect GPAP, data are shared within the Solve-RD consortium via the European Genome-Phenome Archive (EGA) [25]. We retrieved data for our analysis through a cloud-based central database RD3 (rare disease data about data) using the MOLGENIS open-source data platform [26]. RD-Connect GPAP ensures data standardisation, pseudonymisation and harmonisation according to GA4GH-approved standards (Global Alliance for Genomics and Health) [27], in a computer-readable format (Phenopacket) [28] which enables exchange of phenotypic and family information.

### Ontologies used

To ensure data standardization, the Human Phenotype Ontology (HPO) [22], the Orphanet Rare Disease Ontology (ORDO) [23] and HOOM (HPO-ORDO Ontological Module) [24] were used. HPO provides a standardized vocabulary of phenotypic abnormalities encountered in human disease. For this study, we used the HPO 2020-10-12 release (including 15,656 unique HPO terms). ORDO is a structured vocabulary for rare diseases derived from the Orphanet knowledge base including relationships between diseases and between diseases and genes. This study uses ORDO v3.1, Dec 2020 including 9338 active clinical entities. Clinical entities in ORDO are designated in this paper by their unique identifier in Orphanet, the ORPHAcode. HOOM is an ORDO module that qualifies the association between a clinical entity and its HPO-based phenotypic abnormalities according to their frequency. For this study, we used the version 1.5 (Dec 2020) including 1,867,364 ORDO-HPO associations. Data in ORDO and in HOOM are manually curated and expert-validated.

### Data and models employed

#### Study population

**Fig. 1:**
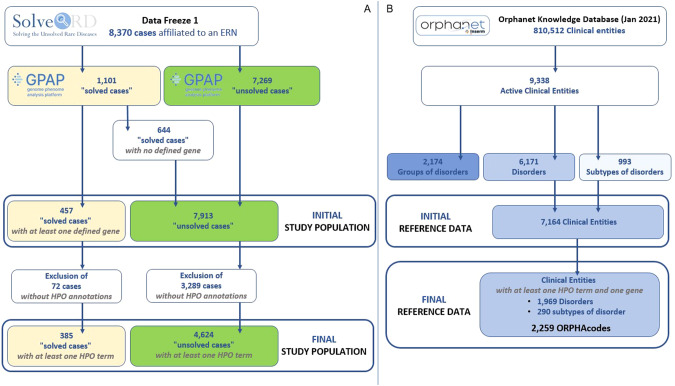
Data filtering workflow for cases and Orphanet data. **A** Filtering of cases from data freeze 1 cohort extraction, definition of the initial population by redefining solved and unsolved cases. Obtention of the final study population after exclusion of cases with no HPO annotation. **B** Filtering of clinical entities of Orphanet database for active clinical entities, preparation of the final reference data (ORPHAcodes).

Solve-RD collects phenomics and genomics data from patient cohorts [7] and releases them as data freezes. The present study uses data selected from the 2020 Data Freeze 1 which contains data (phenotype, pedigree, genotype) of 8370 cases affected by a RD and submitted by the four ERNs [7] to the RD-Connect Genome-Phenome Analysis Platform (GPAP) [21]. This initial dataset contained 1101 “solved cases” (patients annotated as “solved” in the GPAP platform by the data submitter) and 7269 “unsolved cases” (patients annotated as “unsolved” by the data submitter, therefore with an inconclusive whole exome/genome sequencing WES/WGS result). Our study population was built by filtering the cases as depicted in Fig. 1A: (i) 644 cases with no causative gene were removed from the solved cases population and added to the unsolved cases population, as cases without a causative gene are considered unsolved in this analysis; (ii) cases without phenotype annotations that are not suitable for a phenotypic comparison were removed from both populations (72 solved and 3289 unsolved cases). Thus, the resulting final population included 385 solved cases and 4624 unsolved cases (4.6% and 55.2% of the data freeze 1 respectively).

#### Reference data

Reference data is obtained after applying the following filtering to ORDO (Fig. 1B) where “groups of disorders” and “inactive clinical entities” were removed (see Orphanet’s definitions in Additional informations file). Of the resulting active entities (6171 disorders and 993 subtypes of disorder) only entities associated with (a) at least one gene (3789 entities) and (b) at least one HPO term (3955 entities) are kept. Thus, the resulting 2259 entities (ORPHAcodes) which satisfy (a) and (b) served as reference data (31.5% of Orphanet active clinical entities).

### Phenotypic similarity methods/algorithms

During the Solve-RD project, Köhler et al. developed runSolveRD.jar, a single JAR executable file packaging eight methods/algorithms [29–34] capable of computing similarity measures [31]. These methods/algorithms calculate one-to-all ranked similarities between each case with reference RD and also amongst all cases. Similarity scores range from 0 to 1 (where 1 is the closest point): for each case, case-case and case-disorder associations are ranked by decreasing similarity score. For the purpose of this study, from the 8 algorithms provided, we selected Resnik symmetric method due to its best performance [35] and the 50-first results were retrieved, with no limiting similarity thresholds.

### Workflow design

**Fig. 2:**
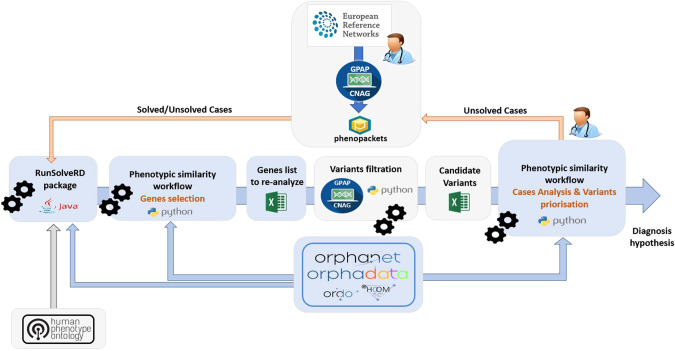
Whole analytic process: Cases are submitted in RD-Connect Genome-Phenome Analysis Platform (GPAP) then processed for phenotypic similarity calculations. From selected genes, variant candidates detected by GPAP after re-analysis and filtration steps are added to phenotypic/genotypic results. Using this data, Cytoscape JS computes networks capable of providing to clinicians a visual interpretation of cases’ clusters.

**Fig. 3:**
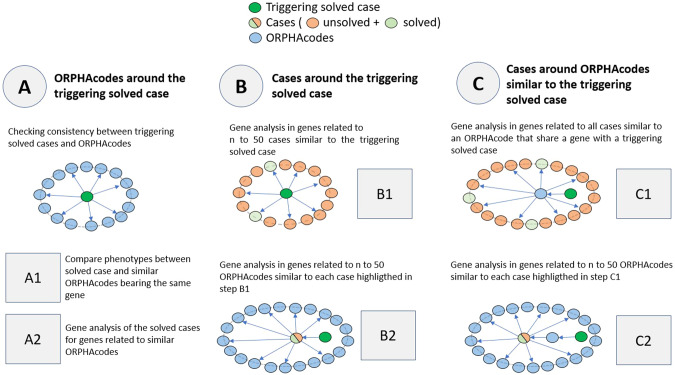
Schema of the three complementary approaches **A**, **B** and **C**. **A**—ORPHAcodes around the triggering case, **B**—Cases around the triggering case and **C**—Cases around ORPHAcodes similar to the triggering case.

As schematized in Fig. 2, after having obtained similarity calculations we applied a workflow which is divided into three complementary approaches A, B and C, each using a triggering case (solved case) as a starting point for the purpose of validating the methodology (Fig. 3). Through in-house Python 3.8 scripts, each approach generates a cluster around each point of interest thus maximizing the one-to-all phenotypic similarity explorations. Each point, which is a case or an ORPHAcode related to the triggering case, can hold genetic information if it exists and a list of genes is submitted to RD-Connect GPAP for identification of candidate variants [8].

#### A—ORPHAcodes around the triggering case

This approach aims to check that the solved case phenotype (i) clinically fits a known RD phenotype (ORPHAcode) retrieved with the 50-first similar ORPHAcodes and (ii) is caused by mutations in the same gene **(A1)**. In case of phenotype inconsistency, the genes associated to the first 50 similar ORPHAcodes are re-analysed for the triggering case (based on data present in RD-Connect GPAP) to eventually detect further candidate pathogenic variants **(A2)**.

#### B—Cases around the triggering case

This approach aims to look for candidate pathogenic variants in the solved case gene in the 50-first similar cases clustered around the triggering case **(B1)**. Variants in genes related to the 50-first similar ORPHAcodes clustered around each case in the cluster are further examined when no variant in the triggering case-related gene is found before **(B2)**.

#### C—Cases around ORPHAcodes similar to the triggering case

This approach aims to look for candidate pathogenic variants in the 50-first similar cases clustered around the ORPHAcode corresponding to the triggering case’s gene. Variants in the triggering case gene are looked for in the cluster **(C1)**, then variants in genes related to the 50-first similar ORPHAcodes around each case within the cluster are further examined when no variant in the triggering case’s gene during stage C1 is found **(C2)**.

### Variant prioritisation methods

Genes retrieved by similarity results in the A, B and C approaches, were analyzed by RD-Connect GPAP using genomic data from all individuals included in the study as described in Matalonga et al. [8]. Genomic data was filtered by (i) rare variants (MAF < 0.01 according to gnomAD and MAF < 0.02 according to RD-Connect GPAP internal frequency), (ii) with a high (truncating) or moderate (amino acid change) impact at the protein level according to Variant Effect Predictor (VEP) from Ensembl and (iii) falling within a gene included in the specific gene list per individual generated using similarity results. Variants were annotated with the RD-Connect GPAP annotations [21] including ClinVar and ACMG (InterVar) clinical significances.

Variants were then filtered based on their clinical significance and only pathogenic and likely-pathogenic variants according to ACMG guidelines [36] were further submitted to referring clinicians for final evaluation and discussion during a multidisciplinary meeting.

### Example case selection

To test the relevance of the designed workflow, four solved cases were selected as triggering cases in order to explore four different situations:

- A triggering case clinically similar to an ORPHAcode associated with the same causative gene (PX_8147689, *KIF5A*-related phenotype).
- A triggering case clinically similar to an ORPHAcode associated with the same causative gene but presenting an unexpected phenotypic variation compared to that of the ORPHAcode (PX_2811577, *SPAST*-related phenotype).
- A triggering case not clinically similar to the expected gene-related ORPHAcode (PX_2354306, *TBL1XR1*-related phenotype).
- A triggering case whose causative gene is associated with more than one similar ORPHAcode (PX_1162604, *CASQ1*-related phenotype).

The results of the 3-approaches workflow triggered by these four cases were submitted to a group of clinicians from participating ERNs for a final evaluation during four workshops. Errors in the phenotypic annotations detected during the discussions were corrected by the clinicians and the whole workflow was re-run for those cases.

## Results

The number of pathogenic or likely pathogenic variants detected is summarized in Table 1.

**Table 1:**
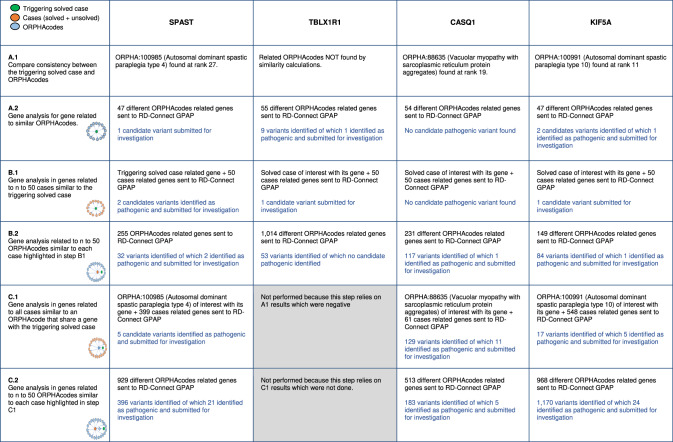
Summarized results of the A, B and C approaches for the 4 triggering selected cases.

The A1 approach is aimed at validating that the phenotypic similarity calculation was able to find the corresponding rare disease, and at checking the clinical consistency between the patient phenotypic description and that of the rare disease, thus confirming the diagnosis. Similarity calculations were able to find the candidate clinical diagnosis in 3 out of 4 cases within the first 30 results (at positions 11, 19 and 27 for *KIF5A*-, *CASQ*1- and *SPAST*-related cases respectively). The *TBL1XR1*-related ORPHAcode (Pierpont syndrome, ORPHA:487825) however was not found in the first 50 results triggered by the *TBL1XR1*-related case, because of its inaccurate phenotypic annotation. After reannotation by the clinician, A1 was re-run and the correct clinical diagnosis was found at rank 15.

When we looked at the clinical consistency between the triggering case and its associated ORPHAcode, the *KIF5A*-related triggering case showed very good clinical consistency with the HPO annotations of the corresponding ORPHAcode Autosomal dominant spastic paraplegia type 10 (ORPHA:100991) [37], subsequently confirmed by the clinician. However, *SPAST*- and *CASQ1*- related triggering cases did not completely correspond to the HPO annotations of their matching ORPHAcodes Therefore, we formulated the hypothesis that a second concurrent genetic anomaly could be at the origin of the cases with atypical phenotypic annotations. The A2 approach performed for the *SPAST*-related case suggested that a homozygous deletion in *LDHA*, causing Glycogen storage disease due to lactate dehydrogenase M-subunit deficiency (ORPHA:284426) [38] deserved further investigation and could contribute to the phenotype of this case, one that could not be diagnosed as a Pure spastic paraplegia type 4 (ORPHA:100985).

As for the *CASQ1* triggering case, only one out of two possible ORPHAcodes associated with *CASQ1* corresponding disorders (ORPHA:88635) was found based on phenotypic similarity. Discussion with clinicians led to the conclusion that, because of lack of perfect consistency between the case and the possible diagnosis, other genetic alterations could be involved in the phenotype. The A2 approach performed for this case retrieved another variant in the *TTN* gene, classified in ClinVar as “conflicting interpretations of pathogenicity”; the case is currently undergoing further investigation.

In conclusion, running phenotypic similarity calculations through approach **A** allowed us to unveil phenotypic description quality issues and highlighted the need for further analysis in order to provide a clinical diagnosis before considering cases as solved.

The B1 approach used information from triggering cases to help solve the unsolved cases in the same phenotypic cluster. Interesting candidate variants were found for three unsolved cases related to *SPAST-*, *TBL1XR1-* and *KIF5A-* triggering cases. A variant was found in an unsolved case related to *CASQ1-*triggering case, but it was not available in ClinVar and was likely an artefact. Another variant was found in a solved case related to the *SPAST-*triggering case, confirming its clinical diagnosis.

After discussion with clinicians, the *KIF5A* candidate variant (c.226 G > C, p.Ala76Pro, (Supplementary Table A and B)) seemed like a promising non-sense variant, even if not available on ClinVar. It is located in the part of the gene coding for the protein motor region and it was found in a case at rank 16 of similarity, with a phenotypic description of pure spastic paraplegia. Despite the fact that the parent’s DNA was not available and no functional analysis could be carried out, this finding will likely solve the case.

The *SPAST* missense variant identified (c.134 C > A;p.Pro45Gln, (Supplementary Table A and B)) in an unsolved case within the *SPAST* cluster bears conflicting evidence of interpretation in reference databases and it does not segregate in the other symptomatic siblings; therefore, this case could not be solved by this approach. The variant found in the *TBL1XR1* gene (c.1184 T > A (p.Tyr395Phe)) for the unsolved case in the *TBL1XR1* case phenotypic cluster did not explain the typical Amyotrophic lateral sclerosis (ALS) phenotype of the case based on the feedback from the clinical expert, although it is described as likely pathogenic, pointing out the need for variant reclassification.

The B2 approach further examines the unsolved cases retrieved in B1 approach, analysing the genes involved in the first 50 phenotypically-related ORPHAcodes of each unsolved case. In both *SPAST*- and *KIF5A*- clusters, a variant of interest (c.136 G > T (p.Asp46Tyr)) was found in the *GALC* gene, known to be causative of Krabbe disease (ORPHA:487) [39]. This could partially explain the case’s phenotype, however additional information on the case’s evolution was not available to confirm or to infirm the diagnostic hypothesis. All the other selected variants were finally discarded because either classified as likely benign or considered not explanatory of the phenotypes by the clinicians.

In conclusion, the **B** approach yielded results were modest but potentially allowed for two unsolved cases to be explained, reconsidering the phenotypes from a new perspective because of the unexpected variants found.

The **C** approach aimed at analysing the clusters around the ORPHAcode related to the gene of the triggering case. The C1 approach was possible for clusters around ORPHAcodes related to all triggering cases except *TBLX1R1*, because there was no ORPHAcode found in the phenotypic cluster at the first analysis (A1) when the workshops were conducted. Five *SPAST* pathogenic variants were found in 5 solved cases clustered around Autosomal spastic paraplegia type 4 (ORPHA:100985) and were therefore considered as positive confirmations of our analysis. Five *KIF5A* candidate variants were found in four unsolved cases in a SPG10 (ORPHA:100991)-centred cluster. Despite their classification as likely pathogenic in ClinVar, they were discarded because they are not in the motor region of *KIF5A* gene, suggesting that they might have been erroneously classed as likely pathogenic in the reference databases. In addition, for one unsolved case clinically consistent with SPG10 phenotype, a variant (c.1373 C > T, (p.Ser458Phe) was identified. This case has been published, and although the variant in *KIF5A* was discussed by the authors, the case was still labelled as unsolved [40]. Indeed, clinicians agreed on the diagnostic hypothesis but suggested that the study of further cases is needed before certifying the variant’s pathogenicity. No variants were found in the *CASQ1*-related ORPHAcodes-centric clusters.

In the C2 approach, each unsolved case belonging to the reference ORPHAcode’s phenotypic cluster, and not explained by C1 approach, is reanalysed for the genes causative of its top 50 most similar ORPHAcodes. In the ORPHA:100991 (SPG10) phenotypic cluster, a pathogenic variant in *VCP*, known to be associated with amyotrophic lateral sclerosis [41] was found for an unsolved case, which appeared consistent with the case’s clinical presentation. As the initial phenotypic description was limited, it was decided to perform the **A** approach after the case was reannotated by the clinicians. ORPHA:803 (ALS) was then found at rank 24 by the similarity calculation. A diagnostic confirmation for this case is expected after the case’s re-examination.

In conclusion, the **C** approach identified a number of candidate variants that triggered re-investigation of cases both from a clinical and molecular point of view.

Overall, the phenotypic similarity workflow initiated with 4 exemplary cases retrieved a total of 725 cases (14.5% of the study population) in the first-50 ranks of the workflow approaches and these were further analysed for variant detection. Variants of interest (pathogenic or likely pathogenic) were found in 64 out of these 725 cases (8.8%) thus leading to the formulation of diagnostic hypotheses. These hypotheses were validated for 42.1% (27/64) of those cases. In 7 cases (10.9%) the diagnostic hypotheses raised were considered as promising by the clinicians, but require additional investigation.

## Discussion

The International Rare Disease Research Consortium (IRDiRC) roadmap 2017-2027 challenged the community with three goals, of which Goal 1: *All patients coming to medical attention with a suspected rare disease will be diagnosed within one year if their disorder is known in the medical literature; all currently undiagnosable individuals will enter a globally coordinated diagnostic and research pipeline* [3]. These “currently undiagnosable individuals” are defined in Solve-RD as those having undergone an inconclusive WES. The approach proposed in this paper consists in a combination of phenotypic similarity calculations and genomic variant prioritization for re-analysis, based on the hypothesis that using structured, standardized information about solved cases (Phenopackets) and about RD (ORPHAcodes), could help raising diagnostic hypothesis. Other large-scale studies, such as for instance the UK 100,000 Genomes Project [42] have previously demonstrated the potential of phenotype‐driven variant prioritization to improve diagnosis in RD [12, 13]. The proposed workflow allows for the exploration of all the clusters emerging from a systematic one-to-all comparison cascade triggered by solved cases. By selecting exemplar cases, and discussing the results with experts participating in the project, we aim to propose in the future a systematic phenomic-genomic analytical pipeline that can be actionable from any node in the phenotypic clusters, and not only from triggering cases. Despite the fact that our study showed promising results, it has some limitations. Firstly, HPO annotations were not complete: all cases which are not phenotypically annotated (40% in the Solve-RD Data Freeze 1) could not be included in similarity computation, even if the case is associated with an identified causative gene. Furthermore, not every ORPHAcode in the Orphanet knowledge database already has phenotypic annotations, as manually curated annotations are a long, ongoing process, and hence some could not be used by similarity calculations. Nevertheless, it is clear that the process can become more effective as the study population grows and phenotypic/genotypic annotations are added and improved.

Secondly, most of RD are multi-systemic disorders, however we have observed that phenotypic annotations are often influenced by the annotating physician’s medical specialty, thus a bias in calculations could be introduced because of missing discriminant HPO terms. Similarly, we have noticed the trend of using a set of “coarse grain” HPO terms to the detriment of lower and more specific HPO terms, compromising the specificity of the case annotation. 26.4% (1320/5009) of the study population were annotated with less than 5 HPO terms, which constitutes a major limitation, since the best Resnik’s performances are obtained for cases annotated with 10 to 40 HPO terms (see Additional informations file). Taken together, these issues underline the need to raise awareness on how good-quality deep phenotyping is important for improving the results of this kind of approach Integration of genomic data is key for clinicians and researchers to evaluate and further validate diagnosis hypotheses coming from similarity results. This is why we decided to integrate results from similarity calculations together with a downstream analysis of the genomic data (WES or WGS) submitted to the project. To enable this type of high-throughput analysis we used big data technologies and built on the programmatic analysis pipeline of the RD-Connect GPAP. This methodology enables the user to rapidly filter genomic data from thousands of datasets thanks to a specific gene list generated for each case and each approach as an output of the similarity calculations. A list of candidate variants based on any pre-defined filtering step is thus produced: in this case rare variants with a high or moderate impact at the protein level. The whole process is scalable and can be automated. Current limitations lie in data interpretation as the list of candidate variants can be fairly long (>10 variants per case) in approaches comparing up to 50 individuals / disease entities. The elevated number of cases assessed made it impossible for clinicians to evaluate all variants, therefore we had to restrict the submission of results to variants classified as likely pathogenic or pathogenic according to ACMG criteria, missing variants of uncertain significance that could be re-classified as pathogenic after expert evaluation.

Variants of interest were identified in 8.8% (64/725) of all cases found by phenotypic similarity calculations, leading to diagnostic hypotheses. Accessibility to cases and family data will, in these cases, be one of the major issues in the validation of the hypothesis provided and thus the final diagnosis of the case. In 46.8% (30/64) of cases, hypotheses were formulated but a conclusion could not be raised for various reasons such as inconsistency between the case’s phenotype and the hypothesis formulated, difficulties in recalling cases, or the detection of misclassified variants. Indeed, misclassifications in ClinVar and/or ACMG guidelines were observed for two variants. We validated our approach by submitting preliminary results of four selected triggering cases to a group of clinicians from ERNs during four workshops, where cases and phenotypic associations were discussed. The combination of the semi-automated variant prioritization pipeline based on phenotypic similarity calculation with the expertise of clinicians from ERNs has underlined the capacity of this methodology to deliver diagnostic hypotheses that clinicians can use to orient their diagnostic process. A tool exploiting this methodology is therefore being developed and will be published in the near future. Finally, during workshops, it also emerged the need to provide a user-friendly tool for visualizing results. Hence, we are developing a Cytoscape JS [43] based tool, named OrphaScape. OrphaScape, that will be the object of a future publication, will be a valuable tool for exploring clusters related to cases and/or ORPHAcodes surrounding a case of interest, and will hopefully help in guiding case selection for diagnosis, investigation, hypothesis and analysis.

### Supplementary information

Additional informations

Description of document

Table A

Table B

## Data Availability

The datasets analysed during the current study are available as phenopackets at the EGA (Datasets EGAD00001009767, EGAD00001009768, EGAD00001009769, and EGAD00001009770, under Solve-RD study EGAS00001003851).
