# Supplementary material for: Phenotypic similarity-based approach for variant prioritization for unsolved rare disease: a preliminary methodological report
Source: Eur J Hum Genet. 2023 Nov 6;32(2):182–9. doi: 10.1038/s41431-023-01486-7 (PMC10853199; doi:10.1038/s41431-023-01486-7)

**Additionnal informations :**

- Cases distribution

Actually there is 24 ERN, launched in 2017, involving more than 900 highly-specialised healthcare units from over 300 hospitals in 26 Member States. More information could be found on the dedicated link free available at:

<https://health.ec.europa.eu/european-reference-networks/networks_en>

The case distribution submitted per ERN after filtering are:

- 2,306 cases by ERN-RND: 94 solved and 2,212 unsolved
- 1,782 by ERN-ITHACA: 228 solved and 1,554 unsolved
- 624 by EURO-NMD: 57 solved and 567 unsolved
- 297 by ERN-GENTURIS: 6 solved and 291 unsolved
- Orphanet definitions

Group of disorders, disorder and subtype of disorders follow Orphanet’s hierarchical levels of the clinical entity that determine the level of precision of each diagnosis included in the nomenclature (see figure below).

A group of disorders is a collection of clinical entities sharing a set of common features.

A disorder is a clinical entity characterised by a set of homogeneous phenotypic abnormalities and evolution allowing a definitive clinical diagnosis.

A subtype of disorder is a subdivision of a disorder according to a positive criterion.


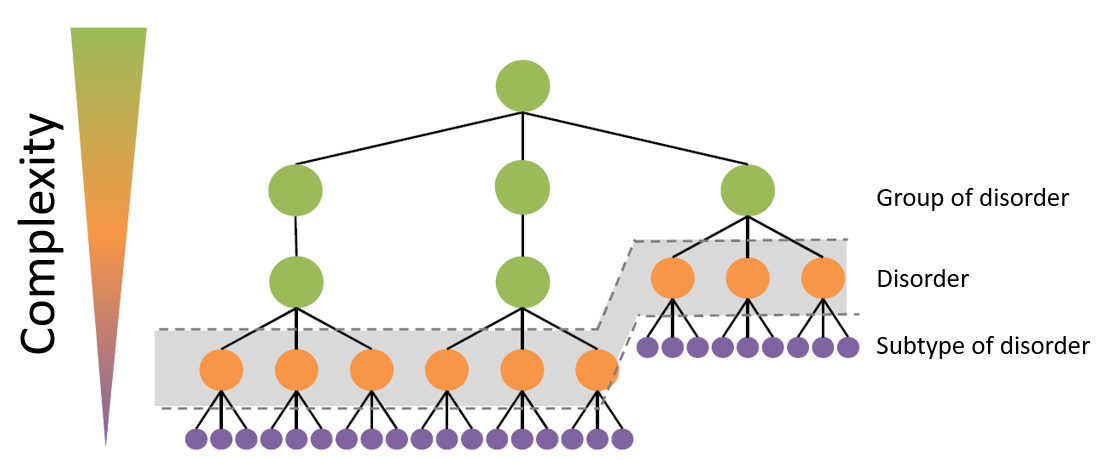


An active entity is a clinical entity compliant to the Orphanet’s nomenclature for direct use in European health coding systems, while an inactive entity is a clinical entity that no longer belongs to the nomenclature and is no longer part of the Orphanet classification. This includes obsolete entities, deprecated entities, and entities that have been inactivated because they are not rare in Europe.

- Distribution of number of HPO terms in solved cases according to their rank in similarity calculations computed by Resnik algorithm.


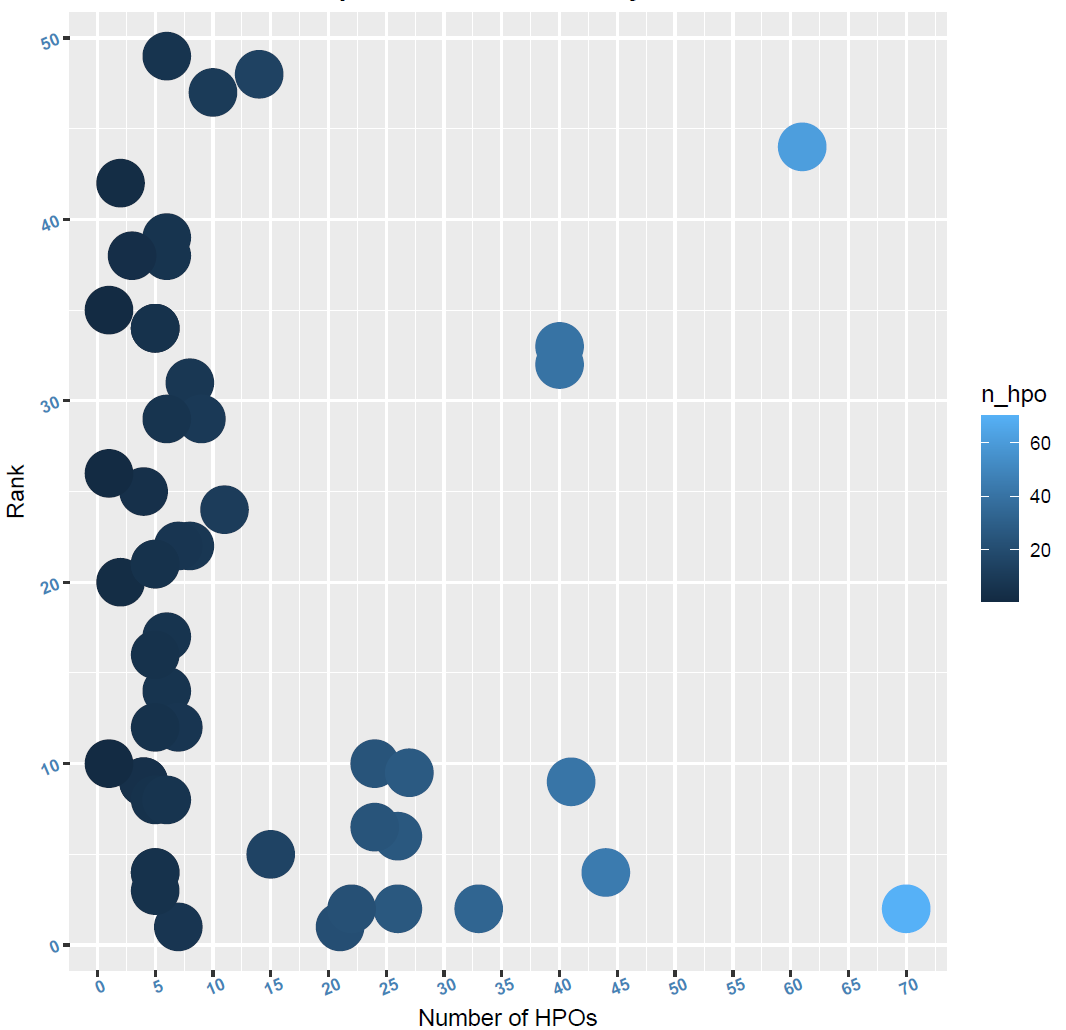

Supplement: Supplementary file 1 — Additional informations [file 41431_2023_1486_MOESM1_ESM.docx]
