# Supplementary material for: Phenotypic similarity-based approach for variant prioritization for unsolved rare disease: a preliminary methodological report
Source: Eur J Hum Genet. 2023 Nov 6;32(2):182–9. doi: 10.1038/s41431-023-01486-7 (PMC10853199; doi:10.1038/s41431-023-01486-7)
Supplement: Supplementary file 2 — Description of document [file 41431_2023_1486_MOESM2_ESM.docx]

**Summary of files:**

**Table A_All steps results** is an excel file with the results of the 4 workshops, containing results of the 4 cases divided in leaf corresponding for each to one of 3 approaches of the methodology, with Orphanet data and variant analysis.

**Table B_Features** description of the variants analyses in excel files is an excel file describing the columns presented in the entire document “all steps”.
